# Epidemic trend of periodontal disease in elderly Chinese population, 1987–2015: a systematic review and meta-analysis

**DOI:** 10.1038/srep45000

**Published:** 2017-03-30

**Authors:** Hongmei Yang, Li Xiao, Lei Zhang, Stacytabi Deepal, Guo Ye, Xiaonan Zhang

**Affiliations:** 1 College of Stomatology, Chongqing Medical University, Chongqing, China; 2 Chongqing Key Laboratory of Oral Diseases and Biomedical Sciences, Chongqing, China; 3 Chongqing Municipal Key Laboratory of Oral Biomedical Engineering of Higher Education, Chongqing, China; 4 Research Center for Public Health, School of Medicine, Tsinghua University, Beijing, 100084, China; 5 Central Clinical School, Faculty of Medicine, Nursing and Health Sciences, Monash University, Melbourne, VIC, Australia; 6 Melbourne Sexual Health Centre, Alfred Health, Melbourne, VIC, Australia

## Abstract

Periodontal disease is a common oral health problem in the elderly population. The prevalence varied substantially due to absence of a universal diagnostic criteria. We conducted a systematic review to identify the epidemiological characteristics of periodontal diseases among Chinese elderly people. A total of 19 articles were included. The pooled detection rates for three indicators, including bleeding on probing (BOP), pocket depth (PD) and clinical attachment loss (CAL), were 53.9% (95% CI: 43.8–63.9%), 57.0% (50.8–63.2%), and 70.1% (65.4–74.8%), respectively. No significant differences in these indicators between urban and rural population. When stratified by gender, BOP (+) detection rates did not show any differences, but the detection rates of PD ≥ 4 mm and CAL ≥ 4 mm were significantly higher in males than in females (59.3% [53.4–65.2%] versus 50.8% [43.5–58.0%], RR_PD_ = 1.13 [1.01–1.26]; 73.8% [70.0–77.7%] versus 65.2% [60.2–70.2%], RR_CAL_ = 1.21 [1.11–1.32]). No statistically significant differences were observed between CAL ≥ 4 mm and PD ≥ 4 mm (RR = 1.12, [0.83–1.50]). A geographical map based on available data during 1987–2015 showed wide variations of periodontal disease across the mainland China. Some factors such as heterogeneity of case definitions, no specific diagnosis of periodontitis, and variable quality of the included studies could affect the final results. Hence, further high-quality epidemiological studies with standardized diagnostic criteria are needed.

Periodontal disease, including gingivitis and destructive periodontitis, is a severe infection in the adjacent periodontal tissue^1^, which has been reported as one of the three major dental diseases suggested by the World Health Organization (WHO)^2^,^3^. A wide spectrum of clinical manifestations includes calculus dentalis, gingival inflammation, periodontal pocket, and attachment loss. It is considered to be one of the major causes of adult tooth loss^4^,^5^,^6^, thereby affecting esthetics and individual’s confidence. Chewing difficulties resulting from the periodontal disease may interfere with the nutrition intake, further affecting the generalized health. Evidence suggests that periodontal disease not only involves local oral periodontal tissue, but has a high degree of association with various systemic diseases, such as diabetes, cardiovascular disease, stroke, preterm low birth-weight newborns, respiratory infections, and bacteremia^7^,^8^. An increasing disease burden of severe periodontitis from 1990 to 2010^9^ warrants our attention due to a growing aged population worldwide.

Prevalence of periodontal disease reported in different countries has shown substantial variability, such as 54.8% in Hungary, 2006^4^; 38.6% in Brazil, 2009^5^; 14.9% in France, 2011^6^; 70% in Kenya, 2012^10^; and 29.4% in America, 2012^11^. A limited number of studies reported the prevalence of periodontal disease in Chinese population until 1980’s. In recent decades, numerous investigations on periodontal diseases have been conducted in different regions of China including two national oral health surveys^12^,^13^. The outcomes have differed across Chinese regions. For instance, the prevalence of periodontal disease was nearly 50% in Beijing^14^, while 81.08% in Henan, as reported by Yang *et al*. in 2005^15^. This discrepancy could be related to different socioeconomic conditions, sample composition, survey time, investigators and sampling methods. More importantly, it is difficult to achieve uniform diagnostic criteria for periodontal disease in an epidemiological investigation, which can substantially affect the investigation outcomes.

China has become an aging society, most likely due to improvement in living standards and the extension of life expectancy. According to sixth national population census in 2010, 118.83 million people were found to be in the age group of 60 and above, making 8.87% of the national population^16^. This population is expected to rise up to 36.5% by 2050, higher than most developed countries^17^. Since periodontal disease is a common oral health problem in the elderly population, it has become a major health issue worthy of attention in China. Therefore, it is important to understand the epidemiological situation of the disease for effective prevention as well as the allocation of health resources.

Given that no universal diagnostic standard is achieved, there have been no nationwide studies on the prevalence of periodontal disease in mainland China. Two national oral health surveys were conducted in 1995 and 2005, which reported different indicators for this disease. To the best of our knowledge, there are no systematic reviews published on the prevalence of periodontal disease up till now, and its epidemiological status among Chinese people remains unclear. Therefore, this study focused not only on the prevalence of periodontal disease, but also its geographical distribution, gender difference, and temporal trends in the elderly Chinese population. For the first time, we quantitatively analyzed the data from all the regional cross-sectional surveys on the periodontal disease among Chinese people aged 60 and above, to explore its epidemiological characteristics in mainland China.

## Results

### Literature search and quality assessment

**Figure 1:**
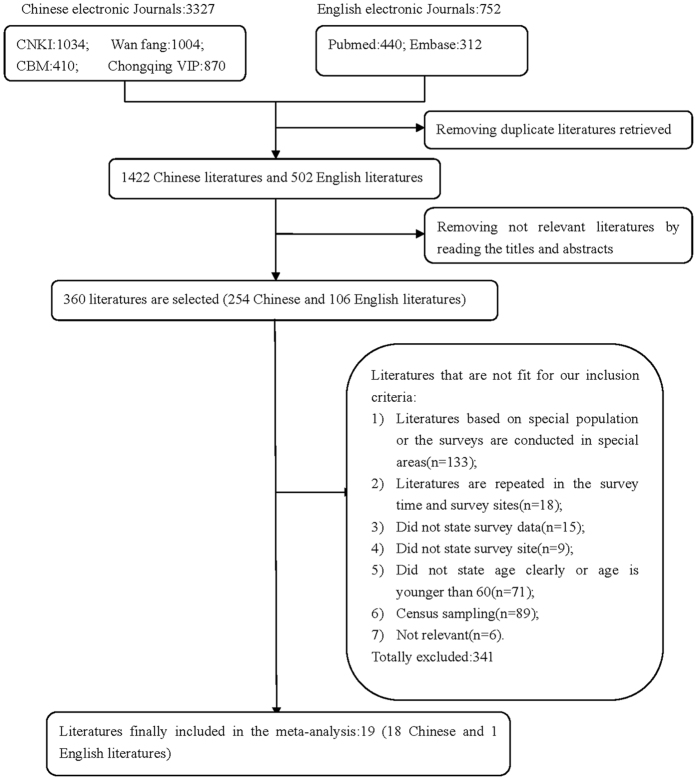
Flow chart of literature search and selection.

A total of 4,079 studies reporting the prevalence of periodontal disease were identified by keyword search on PubMed (n = 440), Embase (n = 312), CNKI (n = 1,034), the WanFang Database (n = 1,004), the Chongqing VIP Database (n = 870), and CBM (n = 410). Subsequently, 2,155 duplicate records were removed from the pooled database and 1,564 irrelevant studies were excluded by screening the relevance of the titles and abstracts. After examining the full texts, 341 studies did not meet the inclusion criteria: 133 studies on special populations or the surveys were conducted in special areas; 18 publications had repeat survey time and sites; 15 did not report survey data; nine did not report survey site; 71 did not report age clearly, or age was younger than 60; 89 did not use random sampling; six did not report relevant information. Finally, 19 studies (18 studies in Chinese and one in English) met the selection criteria for our meta-analysis, including two national-level, four provincial-level, and 13 city-level articles^12^,^13^,^14^,^15^,^18^,^19^,^20^,^21^,^22^,^23^,^24^,^25^,^26^,^27^,^28^,^29^,^30^,^31^,^32^ (Fig. 1). The study selection and data extraction were performed by two authors. The weighted Kappa statistic for inter-examiner consistency was 0.82 during the title and abstract screening, and 0.63 in the full-text analysis (Supplementary Table S1).

The baseline characteristics of the 19 studies are summarized in Table 1. Among these, random sampling was applied in 14 studies, while another five studies did not mention the survey methods. Moreover, 18 surveys recruited dentists, trained examiners, experienced dentists or doctors as investigators. Regarding quality assessment (Supplementary Table S2), the number of affirmative answers (‘yes’) for the 32 listed items/sub-items on the Strobe checklist for each study was at least 25 (range: 25–32), suggesting that the quality of the 19 eligible studies were satisfactory.

As far as diagnostic criteria for the periodontal disease is concerned, different studies adopted different criteria for the diagnosis of periodontal disease. Three studies were based on the criteria suggested by the WHO^33^; five studies adopted the guidelines for the second National Oral Health Survey; ten used the guidelines for the third National Oral Health Survey, and another one survey did not mention the source of diagnostic criteria they used. To sum up, the diagnosis of periodontal disease was primarily based on bleeding on probing (BOP), or pocket depth (PD) in epidemiological surveys performed till the year 2009; subsequently, most surveys reported bleeding on probing (BOP), pocket depth (PD), as well as clinical attachment loss (CAL). Among the 19 included studies, 15 studies adopted Community Periodontal Index of Treatment Needs (CPITN)^34^ or Community Periodontal Index (CPI) recommended by WHO^33^, which meant only 10 index teeth (17 16 1 l 26 27 47 46 31 36 37) were examined; three studies examined all the teeth of participants; one study did not mention about the examination method.

### Indicators of periodontal disease

Since the diagnostic criteria of periodontal disease, especially periodontitis, was not unified in the included 19 studies, it is difficult to obtain the prevalence of periodontal disease or periodontitis. Therefore, we tried to analyze the detection rates of bleeding on probing BOP (+), PD ≥ 4 mm, and CAL ≥ 4 mm respectively, to present the epidemiological status of the periodontal disease.

### Detection rates of BOP (+)

#### BOP(+) detection rate over time

**Figure 2:**
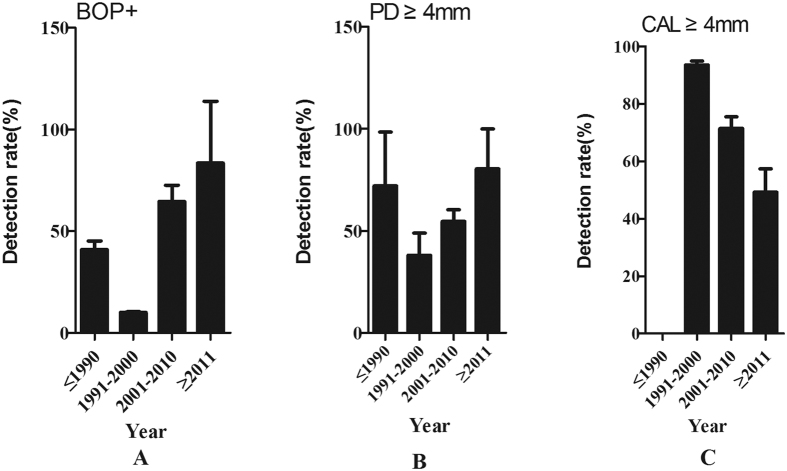
Temporal trends of the detection rates for elderly periodontal disease in mainland China during 1987–2015.

14 of the included 19 studies reported the detection rates of BOP(+). As shown in Table 2, the pooled detection rate was 53.9% (95% CI: 43.8–63.9%). The pooled detection rates of BOP(+) in survey year groups of ≤1990, 1991–2000, 2001–2010 and ≥2011 were 40.8% (95% CI: 36.4%–45.2%), 10.1% (95% CI: 9.8%–10.5%), 64.6% (95% CI: 56.6%–72.5%) and 83.5% (95% CI: 53.1%–113.8%) respectively. Figure 2A showed an overall ascending trend in the estimated BOP(+) detection rates over time in mainland China, while the lowest detection rate was observed in 1991–2000.

#### BOP(+) detection rate by gender

**Figure 3:**
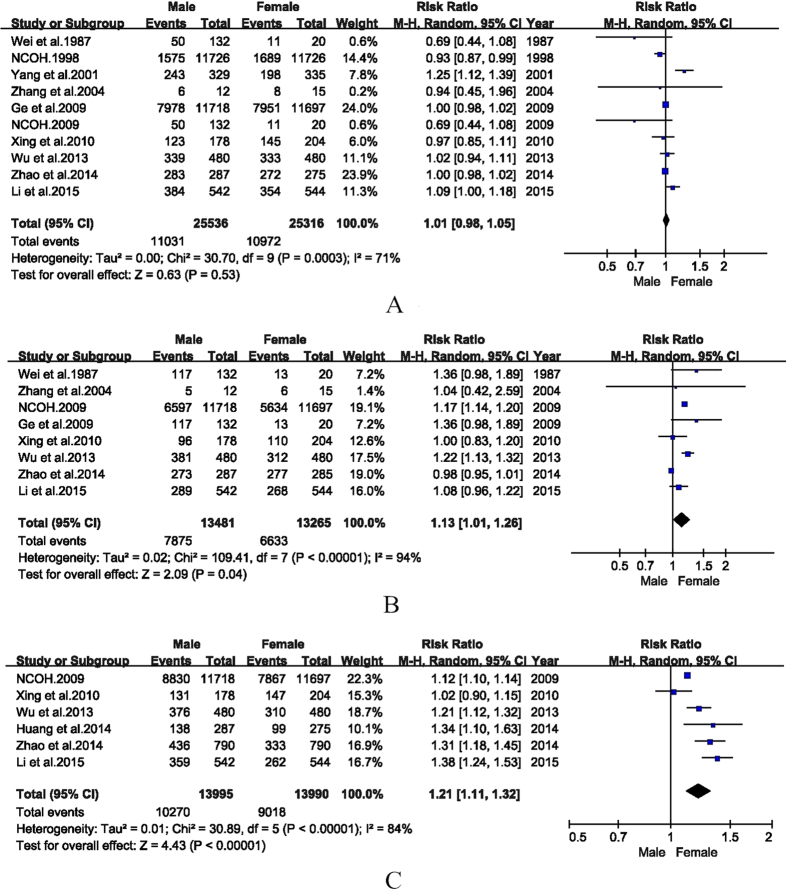
Forest plots of the detection rates for elderly periodontal disease in male and female in mainland China during 1987–2015.

10 articles reported the BOP(+) detection rates for males and females, aged 60–75 years old. The detection rates of BOP(+) in males and females were 55.1% (95% CI: 44.8%–65.5%) and 55.6% (95% CI: 45.0%–66.2%, Table 2), respectively. Besides, no statistically significant difference between males versus females was observed (RR = 1.01, 95% CI: 0.98–1.05, Fig. 3A).

#### *BOP*(+) *detection rate by area*

**Figure 4:**
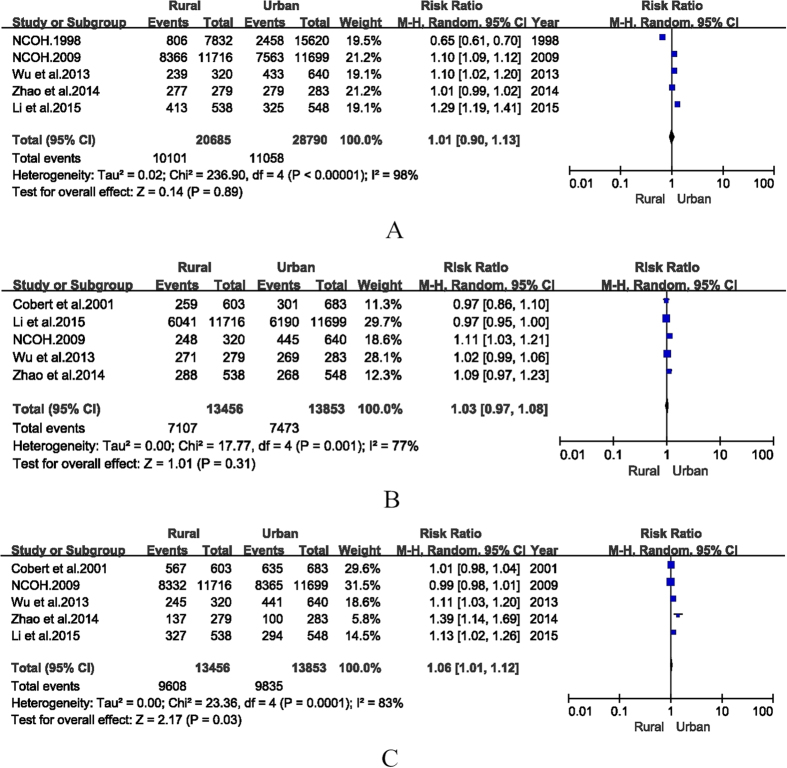
Forest plots of the detection rates for elderly periodontal disease in rural and urban areas of mainland China during 1987–2015.

10 studies provided BOP(+) detection rates in urban areas, while 7 reported in rural areas. The pooled detection rates of BOP(+) in urban and rural China were 52.4% (95% CI: 42.8%–62.0%) and 54.1% (95% CI: 43.1%–65.0%, Table 2), respectively. Only 5 articles stratified BOP(+) detection rates both rural and urban areas. The RR for rural versus urban was 1.01 (95% CI: 0.90–1.13, Fig. 4A).

### Detection rates of PD ≥ 4 mm

#### *PD* ≥ *4* *mm detection rates over time*

A total of 16 articles reported the detection rates of PD during 1987–2015. The pooled detection rate of PD ≥ 4 mm was 57.0% (95% CI: 50.8%–63.2%, Table 2). The detection rates of PD ≥ 4 mm in survey year groups of ≤ 1990, 1991–2000, 2001–2010, and ≥2011 were 72.0% (95% CI: 45.6%–98.5%), 38.0% (95% CI: 27.1%–49.0%), 54.7% (95% CI: 49.1%–60.3%), and 80.4% (95% CI: 60.9%–100.0%), respectively. Further, a substantial ascending trend was observed from 1991 to 2015 (Fig. 2B).

#### *PD* ≥ *4* *mm detection rates by gender*

8 studies reported the PD ≥ 4 mm detection rates for males and females, aged 60–75 years old. The PD ≥ 4 mm detection rates for males and females were 59.3% (95% CI: 53.4%–65.2%) and 50.8% (95% CI: 43.5%–58.0%), respectively (Table 2). Furthermore, the PD ≥ 4 mm detection rate for males was significantly higher than those of females (RR = 1.13, 95% CI: 1.01–1.26, Fig. 3B).

#### *PD* ≥ *4* *mm detection rates by area*

12 studies provided PD ≥ 4 mm detection rates in urban areas, while 7 reported in rural areas. The pooled detection rates of PD ≥ 4 mm in urban and rural China were 57.4% (95% CI: 51.0%–63.8%) and 53.2% (95% CI: 46.4%–60.0%, Table 2), respectively. Only 5 articles reported PD detection rate in the elderly from both urban and rural areas. The RR for rural versus urban was 1.03 (95% CI, 0.97–1.08, Fig. 4B), indicating that there was no significant difference between PD detection rates in urban and rural areas.

### Detection rates of CAL ≥ 4 mm

#### *CAL* ≥ *4* *mm detection rates over time*

7 articles reported the detection rate of CAL ≥ 4 mm during 1987–2015. The pooled detection rate of CAL ≥ 4 mm was 70.1% (95% CI: 65.4%–74.8%, Table 2). The detection rates of CAL ≥ 4 mm during ≤1990 were not available, and the detection rates of CAL ≥ 4 mm in 1991–2000, 2001–2010, and ≥2011 were 93.5% (95% CI: 92.1%–94.8%), 71.4% (95% CI: 67.3%–75.5%) and 49.2% (95% CI: 41.1%–57.3%), respectively. Figure 2C revealed a substantial declining trend during 1991–2015.

#### *CAL* ≥ *4* *mm detection rates by gender*

6 articles stratified detection rates of CAL ≥ 4 mm by gender for the age group 60–75 years. The pooled detection rates of CAL ≥ 4 mm for males and females were 73.8% (95% CI: 70.0%–77.7%) and 65.2% (95% CI: 60.2%–70.2%, Table 2), respectively. The combined detection rate of CAL ≥ 4 mm for males was significantly higher as compared with females (RR = 1.21, 95% CI: 1.11–1.32, Fig. 3C).

#### *CAL* ≥ *4* *mm detection rates by area*

6 articles reported detection rate of CAL ≥ 4 mm in the elderly from urban and rural areas. The pooled detection rates of urban and rural were 69.8% (95% CI: 64.5%–75.1%) and 71.4% (95% CI: 66.7%–76.1%), respectively. Only 5 articles reported detection rate of CAL ≥ 4 mm in the elderly from both urban and rural areas. The RR of CAL for rural versus urban was 1.06 (95% CI: 1.01–1.12, Fig. 4C), indicating that the detection rate in the rural area was slightly higher than that in urban.

### Comparison between detection rates of PD ≥ 4 mm and CAL ≥ 4 mm

6 articles provided both detection rates of PD ≥ 4 mm and CAL ≥ 4 mm. The detection rates of PD ≥ 4 mm and CAL ≥ 4 mm in these 6 articles were 53.8% (95% CI: 47.4%–60.2%) and 70.8% (95% CI: 66.2%–75.3%), respectively. No statistically significant difference was found between the two diagnostic criteria (RR = 1.12, 95% CI: 0.83–1.50, Supplementary Fig. S1).

### Detection rates stratified by the province in mainland China

**Figure 5:**
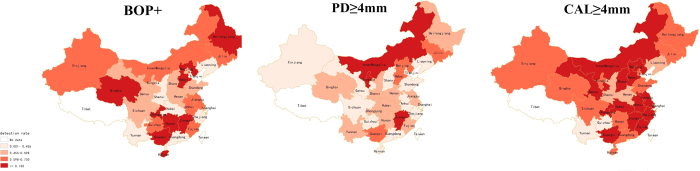
Spatial distribution of the detection rates for elderly periodontal disease in mainland China during 1987–2015 (created by the SuperMap GIS software 2.0).

Figure 5 Shows a color-coded map illustrating the distribution of the detection rates of BOP (+), PD ≥ 4 mm, and CAL ≥ 4 mm in mainland China respectively (data available from most provinces, except Tibet). We created five distribution zones based on the detection rates. The first level represents non-availability of data in the relevant regions. Detection rates range from 0.1% to 45.5%, belong to the second level. The third level represents the detection rates range from 45.5% to 59.8%. The fourth level distribution zone on the map and the detection rates range from 59.8% to 73.0%. The detection rates greater than or equal to 73.0% rank the highest level. Overall, no particular trend in the distribution of BOP (+), PD ≥ 4 mm and CAL ≥ 4 mm detection rates was noticed on the map due to limited information.

### Publication bias and sensitivity test

**Figure 6:**
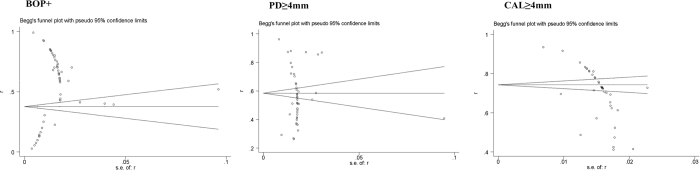
Funnel plots for studies.

Publication bias was observed across the studies in all the three indexes of periodontal disease. The shape of the funnel plots was asymmetric (Fig. 6). Besides, Begg’s tests also indicated the existence of publication bias for all rates (p < 0.001). In a sensitivity analysis, we eliminate one article with the least ‘yes’ responses for quality assessment^4^, the pooled results were not affected, which indicates a statistically robust result.

## Discussion

To our knowledge, this is the first meta-analysis on the prevalence of periodontal disease in mainland China. Our results are combined overall detection rates of BOP (+), PD ≥ 4 mm, and CAL ≥ 4 mm among the elderly, aged 60 years or above in mainland China during 1987–2015. The estimated prevalence of gingivitis was 53.9%, as BOP (+) was the main diagnostic criterion of gingivitis^35^. Since the definition of periodontitis changed over time and diagnostic criteria was various, we estimated the prevalence of periodontitis according to PD ≥ 4 mm or CAL ≥ 4 mm, which was 57.0% and 70.1% respectively.

Periodontal disease includes gingivitis and periodontitis. The definition and diagnostic criteria of periodontitis have been confusing for many years, due to the heterogeneity of clinical presentation. To date, no universally accepted criteria for periodontitis diagnosis, particularly a case definition that can be applied widely in population-based epidemiologic studies, is available. In some surveys, CAL was recorded as an important index for periodontitis. For instance, the prevalence of periodontitis based on CAL ≥ 3 mm was reported to be 69% in Brazil^36^, and 68.6% in America^37^. Additionally, some surveys used three indicators including BOP (+), PD and CAL together to diagnose periodontitis, as a result reporting much lower prevalence, such as 14.9% in French^6^ and 38.6% in Brazil^5^. However, most investigation studies in China only reported the three risk indicators individually, and the prevalence of periodontitis based on PD or CAL alone may overestimate the actual rates. Therefore, this meta-analysis pooled the detection rates (risk indicators) of periodontal disease and performed subgroup analysis on period, gender, geographical locations and diagnostic criteria. Hence, overall estimates of these risk indicators and subgroup analysis were both extensively explored.

CAL was routinely regarded as the gold standard for evaluating the severity and progression of periodontitis^38^. However, given that non-inflammatory gingival recession sites (the healthy periodontal site) may be confused with periodontitis if using CAL alone, some authors recommended that PD and CAL should be united for periodontitis diagnosis^39^. Other scholars suggested^40^,^41^ that the combination of three indexes of BOP (+), PD and CAL should be more accurate, as BOP can reflect the status of gingiva. Considering the diversity of diagnosis criteria for periodontitis, more precise and unified criteria with both high sensitivity and specificity should be explored in the further researches, to report the prevalence of periodontitis.

In studies published before 2009 in China, the diagnosis of periodontitis was mainly based on PD ≥ 4 mm; since then, CAL ≥ 4 mm was added as another important indicator. Although the risk indicators of PD or CAL alone may not necessarily mean the presence of periodontal disease, they could determine the extent and severity of the disease. Previous studies have seldom compared the difference between various diagnostic criteria of periodontitis. In this meta-analysis, no significant difference was found between the two diagnostic criteria of PD ≥ 4 mm and CAL ≥ 4 mm. Nevertheless, some clinician believed that the detection rate of CAL was higher than that of PD, which meant the diagnostic criteria based on CAL was more sensitive than the latter^42^,^43^. The possible discrepancy between the two criteria may not be detected due to the lack of statistical power from a limited number of publications. Hence, more high-quality epidemiologic studies on periodontitis with standardized diagnostic criteria and similar methodology are warranted for further investigations.

In the present meta-analyses, both the detection rates of PD and CAL in males were significantly higher than those in the females, indicating that males are at a higher risk for periodontitis, which is consistent with most studies^44^,^45^. Smoking may be one possible reason for the increased prevalence of periodontitis, since smoking in China is extremely more frequent in males than females in China^46^. Numerous studies have confirmed the positive correlation between smoking and periodontal attachment loss^45^. Moreover, Albandar *et al*.^47^ reported that a higher prevalence of moderate and severe periodontitis, as well as more severe extent of attachment loss and gingival recession were found among smokers.

No significant differences among three indicators for the periodontal disease were observed between urban and rural areas in this meta- analysis. This result was contradicted to several previous studies which suggested that periodontal condition in rural areas is more severe than that in urban^28^,^32^,^45^. Reasons may include the followings: Disparity between urban and rural areas has narrowed with economic growth recently in mainland China^48^. Moreover, the aged people do not pay enough attention to their oral health, even the people in the urban areas. It also mirrors that our affection for the aged is insufficient^49^. Additionally, limited literatures were included in our study to detect the possible difference. Therefore, more epidemiological investigations covering the elderly from both urban and rural areas are needed.

Some potential limitations still exist in our study. Firstly, heterogeneity exists in most meta-analyses, especially in a meta-analysis of epidemiological studies. In our study, the following factors might attribute to the substantial heterogeneity:① large differences existed in sample sizes in the included surveys;② the classification and diagnostic criteria of periodontitis were not standardized in all included studies, which was one of the main heterogeneities of this meta-analysis. Similar heterogeneities can also be seen in previous published meta-analysis^50^,^51^, and subgroup analysis was performed based on same or similar diagnostic criteria to report the results for different diagnostic criteria in the included studies. To address this issue, we combined the detection rates of BOP (+), PD ≥ 4 mm, and CAL ≥ 4 mm respectively, and subgroup analysis based on different diagnostic criteria of periodontitis (PD ≥ 4 mm or CAL ≥ 4 mm) was also performed. No significant difference was observed between the two diagnostic criteria; ③ clinical bias cannot be avoided in an epidemiological investigation like ours. For example, the measurements of CAL and PD were mainly based on examiners’ subjective judgments. Additionally, only index teeth not the full mouth were examined in most surveys. This bias may underestimate the true prevalence given that some teeth with the periodontal disease could be neglected. Secondly, the true prevalence of periodontitis might be overestimated in this meta-analysis, because the included studies only reported the index of PD or CAL individually. Thirdly, since only English and Chinese languages were considered in this meta-analysis, this inherent selection bias could affect the overall estimates. Fourthly, publication bias in three indexes was observed, given that the included studies were sourced from peer-reviewed articles, but not other publication types such as “grey literature.” This bias may also affect the overall estimates.

In conclusion, periodontal disease is a common disease in elderly population in mainland China, with a higher prevalence of periodontitis in males. At present, there are no unified diagnostic criteria for periodontitis in epidemiological surveys. Therefore, further large-scale, high-quality epidemiological studies based on standardized diagnostic criteria are required in the future. Urgent measures are required to improve oral health awareness, and to prevent and control periodontal diseases among the Chinese elder people.

## Methods

### Search strategy

A systematic electronic search was performed by the first and second author with assistance of an expert in epidemiology and statistics, in the following English and Chinese databases: PubMed, Embase, Chinese Biomedical Literature Database (CBM), Chinese National Knowledge Infrastructure Database (CNKI), Chinese Wan Fang Database, and Chongqing VIP database, from the date of establishment till November, 2015. The key terms used in the search included ‘periodontal disease’, ‘prevalence’, ‘epidemiology’, and ‘China’. Moreover, manual searching of reference lists from potentially relevant studies was performed, to identify any additional studies that may have been missed. The systematic review reported here is by the PRISMA (Preferred Reporting Items for Systematic Reviews and Meta-Analyses).

### Study selection and data extraction

The first and second author performed study selection and data extraction independently. In the first-round, non-relevant literature were excluded by screening the titles and abstracts; in the second round, the full-text analysis was performed. Inter-examiner consistency was examined using Cohen’s Kappa^52^. Disagreements were resolved by consensus or by the third author when necessary.

Population-based epidemiological studies published in English or Chinese and available full text, depicting prevalence or indicators for periodontal disease in the elder people aged 60 and above, in mainland China (except for Hong Kong, Taiwan, and Macao), were considered relevant. We excluded studies if they were a review, letters to editors, non-peer-reviewed local or government report, conference abstract or presentation, diagnostic laboratory tests, case reports, master or doctoral theses; studies based on special population (for example, patient-based). Further, exclusion criteria included duplications and studies not reporting geographical locations.

The following information was obtained: first author, year of publication, survey date, interviewer, territorial level, age range, sampling methods, diagnostic criteria, urban vs. rural, total sample size, and total case size. If surveying date was not presented, we assigned it as 2 years before publication.

### Quality assessment

Quality assessment was performed according to the Strengthening the Reporting of Observational Studies in Epidemiology (STROBE) statement (Supplementary Checklist S1), including 22 items (32 sub-items) considered essential for good reporting of observational studies. These items relate to the article’s title and abstract, the introduction, methods, results, and discussion sections and other information. All included articles were assessed for quality by answering ‘yes/no’ for each item/sub-item on the STROBE checklist. For item information that were missing or unavailable in the included papers, such as item 9 (describe any efforts to address potential sources of bias), item 13c (consider use of a flow diagram), and item 22 (funding), we contacted the corresponding authors of the publications for the missing information. The assessment was judged by two independent authors, and disagreements were resolved by consensus or by the third author when necessary.

### Statistical analysis

STATA software version 12.0 was used to calculate the detection rates of BOP (+), PD and CAL (three diagnostic criteria of periodontal disease) and 95% confidence intervals (CI). Q-test and I^2^-statistics were used to explore the source of statistical heterogeneity. When there was no significant heterogeneity (I^2^ < 50% or P > 0.1), a fixed-effects model was selected. Otherwise, the random-effects model was adopted. If there was significant heterogeneity across the studies, subgroup analysis was performed to explore potential factors (gender, location, province, and survey year). Relative Risk (RR) and 95% CI were used to compare differences between different subgroups, using Cochrane Review Manager (RevMan) version 5.1. Furthermore, to reflect the temporal trends of periodontal disease, studies were categorized into four periods: ≤1990, 1991–2000, 2001–2010, and ≥2011. To reflect spatial distribution in mainland China, pooled estimates in each province were entered into the SuperMap GIS software 2.0 to form a prevalence map. Potential publication bias was evaluated by funnel plots and Begg’s test; the result was considered to be significant if p ≤ 0.05. Sensitivity analysis was performed to assess the influence of individual study by the omission of individual studies.

## Additional Information

**How to cite this article:** Yang, H. M. *et al*. Epidemic trend of periodontal disease in elderly Chinese population, 1987–2015: a systematic review and meta-analysis. *Sci. Rep.* **7**, 45000; doi: 10.1038/srep45000 (2017).

**Publisher's note:** Springer Nature remains neutral with regard to jurisdictional claims in published maps and institutional affiliations.

## Supplementary Material

Supplementary Information

## Figures and Tables

**Table 1:** Characteristics of the included 19 studies.

First author& published year	Survey date	Provinces	Territorial levels	U&R	Examination method	Sampling method	Diagnostic criteria	Sample size	BOP(+)		PD > 4 mm		CAL > 4 mm	
									case size	detection rate (%)	case size	detection rate (%)	case size	detection rate (%)
**Wei** ***et al***.^14^	1985	Beijing	provincial	U	CPITN	NA	WHO oral health survey	152	61	40.1	130	85.5	NA	NA
**Chen** ***et al***.^18^	1987	Shanghai	provincial	U	NA	NA	Gingivitis: gingiva inflammation without alveolar bone involved; Periodontitis: true periodontal pocket or odontoseisis.	321	132	41.1	188	58.6	NA	NA
**Cobert** ***et al***.^19^	1997	Guangdong	provincial	U&R	CPI	stratified random	WHO oral health survey	1,286	NA	NA	560	43.6	1,202	93.5
**Yang** ***et al***.^20^	1999	Beijing	city	U	CPITN	random	Guideline for the 2nd National Oral Health Survey	664	441	66.4	215	32.4	NA	NA
**Zhang** ***et al***.^21^	2004	Fujian	city	R	CPITN	Overall	Guideline for the 2nd National Oral Health Survey	27	14	51.9	11	40.7	NA	NA
**Yang** ***et al***.^15^	2002	Henan	city	U	CPITN	random	Guideline for the 2nd National Oral Health Survey	2,320	330	14.2	678	29.2	NA	NA
**Ge** ***et al***.^22^	2008	Hubei	city	U	CPI	NA	Guideline for the 3rd National Oral Health Survey	152	61	40.1	130	85.5	NA	NA
**Xing** ***et al***.^23^	2008	Beijing	city	U	CPI	NA	Guideline for the 3rd National Oral Health Survey	382	268	70.2	206	53.9	278	72.8
**Zhou** ***et al***.^24^	2008	Guangdong	city	U	CPITN	random	Guideline for the 2nd National Oral Health Survey	122	48	39.3	106	86.9	NA	NA
**Dong** ***et al***.^25^	2010	Ningxia	city	U&R	All the teeth	random	Guideline for the 3rd National Oral Health Survey	514	303	58.9	452	87.9	NA	NA
**Li** ***et al***.^26^	2011	Beijing	provincial	U	All the teeth	random	WHO oral health survey	302	NA	NA	183	60.6	NA	NA
**Tian** ***et al***.^27^	2012	Ningxia	city	U	CPI	random	Guideline for the 3rd National Oral Health Survey	687	NA	NA	599	87.2	NA	NA
**Wu** ***et al***.^28^	2010–2012	Hebei	city	U&R	CPI	random	Guideline for the 3rd National Oral Health Survey	960	672	70.0	693	72.2	686	71.5
**Zhao** ***et al***.^29^	2013	Henan	city	U&R	All the teeth	random	Guideline for the 3rd National Oral Health Survey	562	556	98.9	540	96.1	237	42.2
**Huang** ***et al***.^30^	2013	Guangdong	city	U	CPI	random	Guideline for the 3rd National Oral Health Survey	1,580	NA	NA	1,094	69.2	769	48.7
**Zhao** ***et al***.^31^	2012	Yunnan	city	U	CPI	random	Guideline for the 3rd National Oral Health Survey	226	NA	NA	197	87.2	NA	NA
**Li** ***et al***.^32^	2012	Shandong	city	U&R	CPI	random	Guideline for the 3rd National Oral Health Survey	1,086	738	68.0	556	51.2	621	57.2
**NCOH**.**1998**^12^	1995	China	country	U&R	CPI	multi-stage stratified cluster	Guideline for the 2nd National Oral Health Survey	23,452	3,264	13.9	5,217	22.2	NA	NA
**NCOH**.**2009**^13^	2005	China	country	U&R	CPI	multi-stage stratified cluster	Guideline for the 3rd National Oral Health Survey	23,417	15,929	68.0	12,231	52.2	16,697	71.3

NCOH: National Committee for Oral Health; NA: not available; U: urban; R: rural; WHO: World Health Organization; BOP: bleeding on probing; PD: pocket depth; CAL: clinical attachment loss.

**Table 2:** Pooled detection rates of periodontal disease among elderly population in mainland China during 1985–2015.

	BOP(+)				PD ≥ 4 mm				CAL ≥ 4 mm			
	Number of surveys	Sample size	cases	Prevalence (%) [95% CI]	Number of surveys	sample size	cases	Prevalence (%) [95% CI]	Number of surveys	sample size	cases	Prevalence (%) [95% CI]
**Overall**	14	54,129	22,817	53.9[43.8–63.9]	16	32,876	17,492	57.0[50.8–63.2]	7	29,281	20,490	70.1[65.4–74.8]
**Location**												
Urban	10	30,201	11,801	52.4[42.8–62.0]	12	16,197	9,308	57.4[51.0–63.8]	6	15,433	10,604	69.8[64.5–75.1]
Rural	7	21,094	10,383	54.1[43.1–65.0]	7	13,865	7,324	53.2[46.4–60.0]	6	13,838	9,886	71.4[66.7–76.1]
**Gender**												
Male	10	25,536	11,031	55.1[44.8–65.5]	8	13,481	7,875	59.3[53.4–65.2]	6	13,995	10,270	73.8[70.0–77.7]
Female	10	25,316	10,972	55.6[45.0–66.2]	8	13,265	6,633	50.8[43.5–58.0]	6	13,990	9,018	65.2[60.2–70.2]
**Time periods**												
≤1990	2	473	193	40.8 [36.4–45.2]	2	473	318	72.0[45.6–98.5]	0	NA	NA	NA
1991–2000	2	24,116	3,705	10.1[9.8–10.5]	2	1,950	775	38.0[27.1–49.0]	1	1,286	1,202	93.5[92.1–94.8]
2001–2010	8	27,892	17,625	64.6[56.6–72.5]	8	27,892	14,507	54.7[49.1–60.3]	3	24,757	17,661	71.4[67.3–75.5]
≥2011	2	1,648	1,294	83.5[53.1–113.8]	4	2,561	1,892	80.4[60.9–100.0]	3	3,238	1,627	49.2[41.1–57.3]

BOP: bleeding on probing; PD: pocket depth; CAL: clinical attachment loss; CI: confidence interval; NA: not available.
