# Supplementary material for: Epidemic trend of periodontal disease in elderly Chinese population, 1987–2015: a systematic review and meta-analysis
Source: Sci Rep. 2017 Mar 30;7:45000. doi: 10.1038/srep45000 (PMC5372169; doi:10.1038/srep45000)
Supplement: Supplementary Information [file srep45000-s1.pdf]

Epidemic trend of periodontal disease in elderly Chinese population, 1987–2015: a systematic review and meta-analysis

Li Xiao[1,2,3§], Hongmei Yang[1,2,3§], Lei Zhang [4,5,6], Stacytabi Deepal[1,2], Guo Ye[1,2,3], Xiaonan Zhang[1,2,3*]

[1]College of Stomatology, Chongqing Medical University, Chongqing, China

[2]Chongqing key Laboratory of Oral Diseases and Biomedical Sciences, Chongqing, China

[3]Chongqing Municipal Key Laboratory of Oral Biomedical Engineering of Higher Education, Chongqing, China

[4]Research Center for Public Health, School of Medicine, Tsinghua University, Beijing, 100084, China

[5]Central Clinical School, Faculty of Medicine, Nursing and Health Sciences, Monash University, Melbourne, VIC, Australia.

[6]Melbourne Sexual Health Centre, Alfred Health, Melbourne, VIC, Australia.

[§]First co-authors

[*]Corresponding author

Correspondence to:

Xiaonan Zhang, College of Stomatology, Chongqing Medical University, Chongqing, China, No.426 Songshibei Road, Yubei District, Chongqing, China; Tel: +86 23 89035721; Fax: +86 23 89035721; E-mail: pearl-zhang@163.com

Table S1 | Agreement among authors [kappa coefficients]

Table S1a|Agreement among authors on titles and abstracts [kappa coefficients]

		Author2		Total
		Exclusion	Inclusion	
Author1	Exclusion	3646	74	3720
	Inclusion	53	324	377
	Total	3699	398	4097

K1=0.819207

Table S1b|Agreement among authors on full-text analysis [kappa coefficients]

		Author2		Total
		Exclusion	Inclusion	
Author1	Exclusion	329	9	338
	Inclusion	7	15	22
	Total	336	24	360

K2=0.628435

Fig.S1 Forest plot of the detection rates for CAL versus PD in elderly periodontal disease of mainland China during 1987-2015

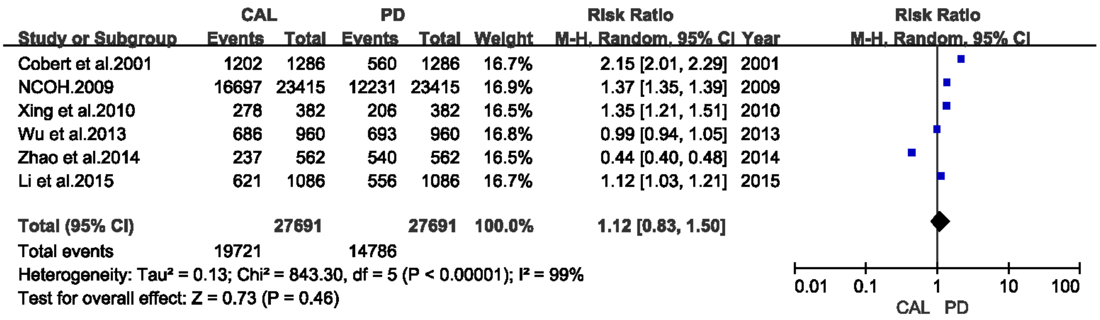

Table S2a | Quality assessment of included 19 studies

First author& published year	Title and abstract		Introduction		Methods													Results										Discussion				Other Information	Number of 'Y
	1a	1b	2	3	4	5	6	7	8	9	10	11	12a	12b	12c	12d	12e	13a	13b	13c	14a	14b	15	16a	16b	16c	17	18	19	20	21	22	
**Wei et al.1987**	Y	N	N	N	Y	Y	Y	Y	Y	Y	Y	Y	N	Y	Y	N	Y	Y	Y	Y	Y	Y	Y	N	Y	N	Y	Y	Y	Y	Y	Y	**25**
**Chen et al.1989**	Y	N	Y	N	Y	Y	Y	Y	Y	Y	Y	Y	N	Y	Y	N	Y	Y	Y	Y	Y	Y	Y	N	Y	N	Y	Y	Y	Y	Y	Y	**26**
**Cobert et al.2001**	Y	Y	Y	Y	Y	Y	Y	Y	N	Y	Y	Y	Y	Y	Y	N	Y	Y	Y	Y	Y	Y	Y	Y	Y	Y	Y	Y	N	Y	Y	Y	**29**
**Yang et al.2001**	Y	Y	Y	Y	Y	Y	Y	Y	N	Y	Y	Y	Y	N	Y	Y	Y	Y	Y	Y	Y	Y	Y	Y	Y	N	Y	Y	N	Y	Y	Y	**28**
**Zhang et al.2004**	Y	Y	Y	Y	Y	Y	Y	Y	Y	Y	N	Y	N	Y	Y	Y	Y	Y	Y	Y	Y	Y	Y	Y	Y	N	Y	Y	Y	Y	Y	Y	**29**
**Yang et al.2005**	Y	Y	Y	Y	Y	Y	Y	Y	Y	Y	Y	Y	Y	Y	Y	Y	Y	Y	Y	Y	Y	Y	Y	Y	N	Y	Y	Y	N	Y	Y	Y	**30**
**Ge et al.2009**	Y	Y	Y	Y	Y	Y	Y	Y	Y	Y	Y	Y	N	Y	Y	N	Y	Y	Y	Y	Y	Y	Y	N	Y	Y	Y	Y	Y	Y	Y	Y	**29**
**Xing et al.2010**	Y	Y	Y	Y	Y	Y	Y	Y	N	Y	Y	Y	N	Y	Y	N	Y	Y	Y	Y	Y	Y	Y	Y	Y	N	Y	Y	N	Y	Y	Y	**27**
**Zhou et al.2010**	Y	Y	Y	Y	Y	Y	Y	Y	N	Y	Y	Y	Y	Y	Y	N	Y	Y	Y	Y	Y	Y	Y	Y	Y	Y	Y	Y	Y	Y	Y	Y	**30**
**Dong et al.2011**	Y	Y	Y	Y	Y	Y	Y	Y	N	Y	Y	Y	Y	Y	Y	N	Y	Y	Y	Y	Y	Y	Y	Y	Y	Y	Y	Y	Y	Y	Y	Y	**30**
**Li et al.2012**	Y	Y	Y	Y	Y	Y	Y	Y	Y	Y	Y	Y	Y	Y	Y	Y	Y	Y	Y	Y	Y	Y	Y	Y	Y	N	Y	Y	Y	Y	Y	Y	**31**
**Tian et al.2013**	Y	Y	Y	Y	Y	Y	Y	Y	Y	Y	Y	Y	Y	Y	Y	Y	Y	Y	Y	Y	Y	Y	Y	Y	Y	N	Y	Y	N	Y	Y	Y	**30**
**Wu et al.2013**	Y	Y	Y	Y	Y	Y	Y	N	Y	Y	Y	Y	N	Y	Y	Y	Y	Y	Y	Y	Y	Y	Y	Y	Y	N	Y	Y	N	Y	Y	Y	**28**
**Zhao et al.2014**	Y	Y	Y	Y	Y	Y	Y	Y	N	Y	Y	Y	Y	Y	Y	Y	Y	Y	Y	Y	Y	Y	Y	Y	Y	N	Y	Y	N	Y	Y	Y	**29**
**Huang et al.2014**	Y	Y	Y	Y	Y	Y	Y	Y	N	Y	Y	Y	N	Y	Y	Y	Y	Y	Y	Y	Y	Y	Y	Y	Y	N	Y	Y	N	Y	Y	Y	**28**
**Zhao et al.2014**	Y	Y	Y	Y	Y	Y	Y	Y	N	Y	Y	Y	Y	Y	Y	Y	Y	Y	Y	Y	Y	Y	Y	Y	Y	N	Y	Y	N	Y	Y	Y	**29**
**Li et al.2015**	Y	Y	Y	Y	Y	Y	Y	Y	N	Y	Y	Y	Y	Y	Y	Y	Y	Y	Y	Y	Y	Y	Y	Y	Y	N	Y	Y	N	Y	Y	Y	**29**
**NCOH.1998**	Y	Y	Y	Y	Y	Y	Y	Y	Y	Y	Y	Y	Y	Y	Y	Y	Y	Y	Y	Y	Y	Y	Y	Y	Y	Y	Y	Y	Y	Y	Y	Y	**32**
**NCOH.2009**	Y	Y	Y	Y	Y	Y	Y	Y	Y	Y	Y	Y	Y	Y	Y	Y	Y	Y	Y	Y	Y	Y	Y	Y	Y	Y	Y	Y	Y	Y	Y	Y	**32**

Y: Yes; N: No; NCOH: National Committee for Oral Health

Checklist S1. **STROBE Statement—Checklist of items that should be included in reports of cross-sectional studies**

	Item No	Recommendation
**Title and abstract**	1	(*a*) Indicate the study's design with a commonly used term in the title or the abstract
		(*b*) Provide in the abstract an informative and balanced summary of what was done and what was found
**Introduction**		
Background/rationale	2	Explain the scientific background and rationale for the investigation being reported
Objectives	3	State specific objectives, including any prespecified hypotheses
**Methods**		
Study design	4	Present key elements of study design early in the paper
Setting	5	Describe the setting, locations, and relevant dates, including periods of recruitment, exposure, follow-up, and data collection
Participants	6	(*a*) Give the eligibility criteria, and the sources and methods of selection of participants
Variables	7	Clearly define all outcomes, exposures, predictors, potential confounders, and effect modifiers. Give diagnostic criteria, if applicable
Data sources/ measurement	8*	For each variable of interest, give sources of data and details of methods of assessment (measurement). Describe comparability of assessment methods if there is more than one group
Bias	9	Describe any efforts to address potential sources of bias
Study size	10	Explain how the study size was arrived at
Quantitative variables	11	Explain how quantitative variables were handled in the analyses. If applicable, describe which groupings were chosen and why
Statistical methods	12	(*a*) Describe all statistical methods, including those used to control for confounding
		(*b*) Describe any methods used to examine subgroups and interactions
		(*c*) Explain how missing data were addressed
		(*d*) If applicable, describe analytical methods taking account of sampling strategy
		(*e*) Describe any sensitivity analyses
**Results**		
Participants	13*	(a) Report numbers of individuals at each stage of study—eg numbers potentially eligible, examined for eligibility, confirmed eligible, included in the study, completing follow-up, and analysed
		(b) Give reasons for non-participation at each stage
		(c) Consider use of a flow diagram
Descriptive data	14*	(a) Give characteristics of study participants (eg demographic, clinical, social) and information on exposures and potential confounders
		(b) Indicate number of participants with missing data for each variable of interest
Outcome data	15*	Report numbers of outcome events or summary measures
Main results	16	(*a*) Give unadjusted estimates and, if applicable, confounder-adjusted estimates and their precision (eg, 95% confidence interval). Make clear which confounders were adjusted for and why they were included
		(*b*) Report category boundaries when continuous variables were categorized
		(*c*) If relevant, consider translating estimates of relative risk into absolute risk for a meaningful time period
Other analyses	17	Report other analyses done—eg analyses of subgroups and interactions, and sensitivity analyses

**Discussion**		
Key results	18	Summarise key results with reference to study objectives
Limitations	19	Discuss limitations of the study, taking into account sources of potential bias or imprecision. Discuss both direction and magnitude of any potential bias
Interpretation	20	Give a cautious overall interpretation of results considering objectives, limitations, multiplicity of analyses, results from similar studies, and other relevant evidence
Generalisability	21	Discuss the generalisability (external validity) of the study results
**Other information**		
Funding	22	Give the source of funding and the role of the funders for the present study and, if applicable, for the original study on which the present article is based

*Give information separately for exposed and unexposed groups.

Note: An Explanation and Elaboration article discusses each checklist item and gives methodological background and published examples of transparent reporting. The STROBE checklist is best used in conjunction with this article (freely available on the Web sites of PLoS Medicine at http://www.plosmedicine.org/, Annals of Internal Medicine at http://www.annals.org/, and Epidemiology at http://www.epidem.com/). Information on the STROBE Initiative is available at www.strobe-statement.org.
